# CD69 serves as a potential diagnostic and prognostic biomarker for hepatocellular carcinoma

**DOI:** 10.1038/s41598-023-34261-1

**Published:** 2023-05-08

**Authors:** Kaihua Tang, Xiaoting Li, Jianwen Mo, Yixuan Chen, Chengyu Huang, Ting Li, Tianjian Luo, Zhijian Zhong, Yongqiang Jiang, Dengfeng Yang, Weiliang Mo

**Affiliations:** 1 Department of Basic Science, YuanDong International Academy of Life Sciences, Hong Kong, 999077 China; 2 Biology Institute, Guangxi Academy of Sciences, Nanning, 530007 Guangxi China; 3 Guangxi Key Laboratory of Marine Natural Products and Combinatorial Biosynthesis Chemistry, Guangxi Beibu Gulf Marine Research Center, Guangxi Academy of Sciences, Nanning, 530007 Guangxi China

**Keywords:** Cell death, Biomarkers

## Abstract

The prevalence and mortality of hepatocellular carcinoma (HCC) are still increasing. This study aimed to identify potential therapeutic targets related to patient prognosis. Data were downloaded from TCGA, GSE25097, GSE36376, and GSE76427 datasets. Differential analysis and enrichment analysis were performed in HCC. Cell deaths were evaluated, and least absolute shrinkage and selection operator regression (LASSO) regression was analyzed to screen candidate genes. Additionally, immune cell infiltration in HCC was assessed. We identified 4088 common DEGs with the same direction of differential expression in all four datasets, they were mainly enriched in immunoinflammation and cell cycle pathways. Apoptosis was significantly suppressed in HCC in GSEA and GSVA. After LASSO regression analysis, we screened CD69, CDC25B, MGMT, TOP2A, and TXNIP as candidate genes. Among them, CD69 significantly influenced the overall survival of HCC patients in both TCGA and GSE76427. CD69 may be a protective factor for outcome of HCC patients. In addition, CD69 was positive correlation with T cells and CD3E. CD69, CDC25B, MGMT, TOP2A, and TXNIP were potential diagnostic and prognostic target for HCC, especially CD69.

## Introduction

Hepatocellular carcinoma (HCC) is the sixth most common malignancy worldwide, with a 5-year survival rate of only 3%, which poses a serious threat to human health and life^1^. The incidence of liver cancer is increasing worldwide, and it is predicted that by 2025, more than 1 million newly diagnosed HCC cases will occur annually^2^. Therefore, it is crucial to find effective diagnostic screening markers and more effective therapies to improve the long-term survival and treatment rates of HCC patients^3^.

Liver resection, liver transplantation, and radiation are helpful treatments for early or intermediate stage if HCC that improve outcomes. Nonetheless, almost 70% HCC patients experience recurrence within 5 years after patients treated with radical surgery or ablation^4^. Advanced HCC is resistant to chemotherapy and radiotherapy, which limit the available treatment options for these patients^5^. 2020 marks the beginning of a third era dominated by combination therapies involving immunotherapy. The life expectancy of HCC patients has improved with the implementation of targeted therapy and immunotherapy^6^. Recently, primary immune checkpoint inhibition has been developed as an effective anticancer strategy. However, most of HCC patients are diagnosed at incurable stages, and the therapeutic means have been challenged^7^. So far, the indications, side effects, and impact on long-term outcomes of chemoprophylaxis and adjuvant therapy remain controversial^8^.

The identification of optimal treatment options, personalized therapy as well as rational multidisciplinary interventions are the directions of recent efforts. The pathogenesis of HCC is more complex, and a deep understanding of the molecular mechanisms underlying the pathogenesis of HCC can provide effective therapeutic strategies to improve the survival rate of patients with HCC^9^. Current studies have found that cell deaths are important for inflammatory diseases^10^. Cell deaths promote inflammation, and fibrosis, targeting them in incurable stage of HCC may represent a therapeutic approach to limit tumor growth^11^. Cell deaths, including necroptosis, cuproptosis, apoptosis, pyroptosis, autophagy, and ferroptosis, may contribute differently to the development of HCC^12,13^.

Here, we not only applied bioinformatics to evaluate the underlying molecular deregulation mechanisms in HCC patients, but also evaluated biomarkers in cell death related genes that play a necessary role in the poor prognosis.

## Material and methods

### Data source and processing

**Figure 1:**
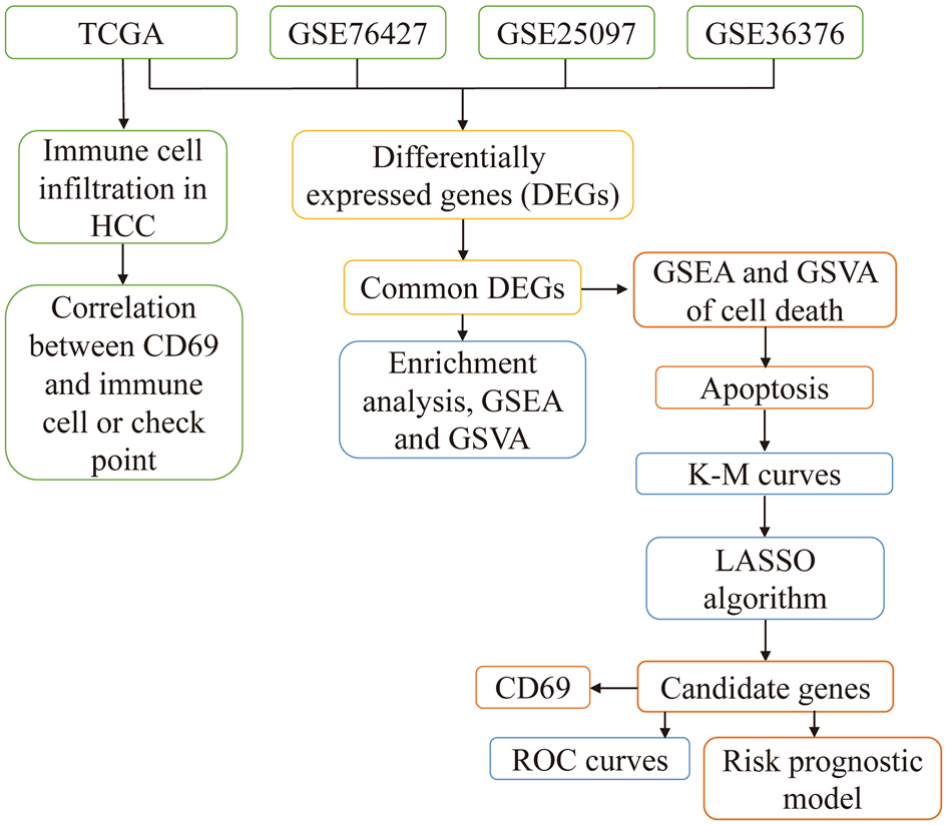
The flowchart of this study. *GSEA* gene set enrichment analysis, *GSVA* gene set variation analysis, *HCC* hepatocellular carcinoma, *K–M* Kaplan–Meier, *LASSO* least absolute shrinkage and selection operator regression, *ROC* receiver operating characteristic curves.

Study design of this study is shown in Fig. 1.

This study included the gene expression profiles of 369 tumor tissue and 50 matched control liver tissue from The Cancer Genome Atlas (TCGA; https://portal.gdc.cancer.gov/) database. Data were normalized with the DESeq2 package in R^14^. In addition, we also collected gene expression profiles of HCC from Gene Expression Omnibus (GEO; https://www.ncbi.nlm.nih.gov/gds/) database. In which, GSE25097 dataset^15^ included 268 tumor and 249 control liver tissue. Data were normalized with the Affy package (v1.76.0) in R^16^. GSE36376 dataset^17^ included 240 tumor and 193 non-tumor liver tissue. Data were normalized with the Lumi package (v2.50.0) in R^18^. GSE76427 dataset^19^ included 115 tumor and 52 adjacent non-tumor liver tissue. Data were normalized with the Lumi package in R.

### Identification of differentially expressed genes and biological functions

Differential analysis for HCC and controls were performed to identify differentially expressed genes (DEGs) using limma package (v3.54.2) in R^20^ for datasets in GEO and using DESeq2 (v1.38.3) package in R^21^ for TCGA. A P < 0.05 was set to screen for statistically significant DEGs. DEGs with the same direction of expression were screened as common DEGs among the four datasets. Kyoto Encyclopedia of Genes and Genomes (KEGG)^22^ and Gene Ontology (GO) were enriched using clusterProfiler package (v4.7.1) in R^23^. P < 0.05 was the screening threshold.

### Assessment of cell death

The gene set enrichment analysis (GSEA)^24^ was used to assess activation or inhibition situations of cell death in HCC. GSEA was performed using clusterProfiler package in R. The levels of cell death were investigated using gene set variation analysis (GSVA, v1.46.0)^25^ between HCC and controls. Cell deaths related genes affecting the overall survival (OS) in TCGA for HCC patients were analyzed using Kaplan–Meier (K–M) analysis.

### Screening of candidate genes

The least absolute shrinkage and selection operator (LASSO) regression was analyzed for genes of cell death significantly affecting OS using glmnet package in R^26^. Then candidate genes were screened with non-zero coefficient by choosing an optimal λ in tenfold cross validation with a minimum cross validation error. The forest plot was constructed using forestplot package in R. Additionally, nomogram was then performed using the rms package (v6.5-0) in R. The area under the receiver operating characteristic curve (AUC) values were calculated using pROC package (v1.18.0) in R^27^. The univariate Cox regression analysis was used to divide HCC samples into low- and high-risk groups based on the median of risk score in TCGA dataset.

### Detection of immune cell infiltration and checkpoints

The CIBERSORT (https://cibersort.stanford.edu/) was employed to analyze the abundance of immune cells for HCC. Single‐sample GSEA (ssGSEA) was performed to determine the infiltration levels for immune cells in HCC using GSVA package (v1.46.0) in R^25^. Correlation between genes and immune cells or between genes and immune check points were determine using Pearson correlation.

## Results

### Differentially expressed genes in HCC

**Figure 2:**
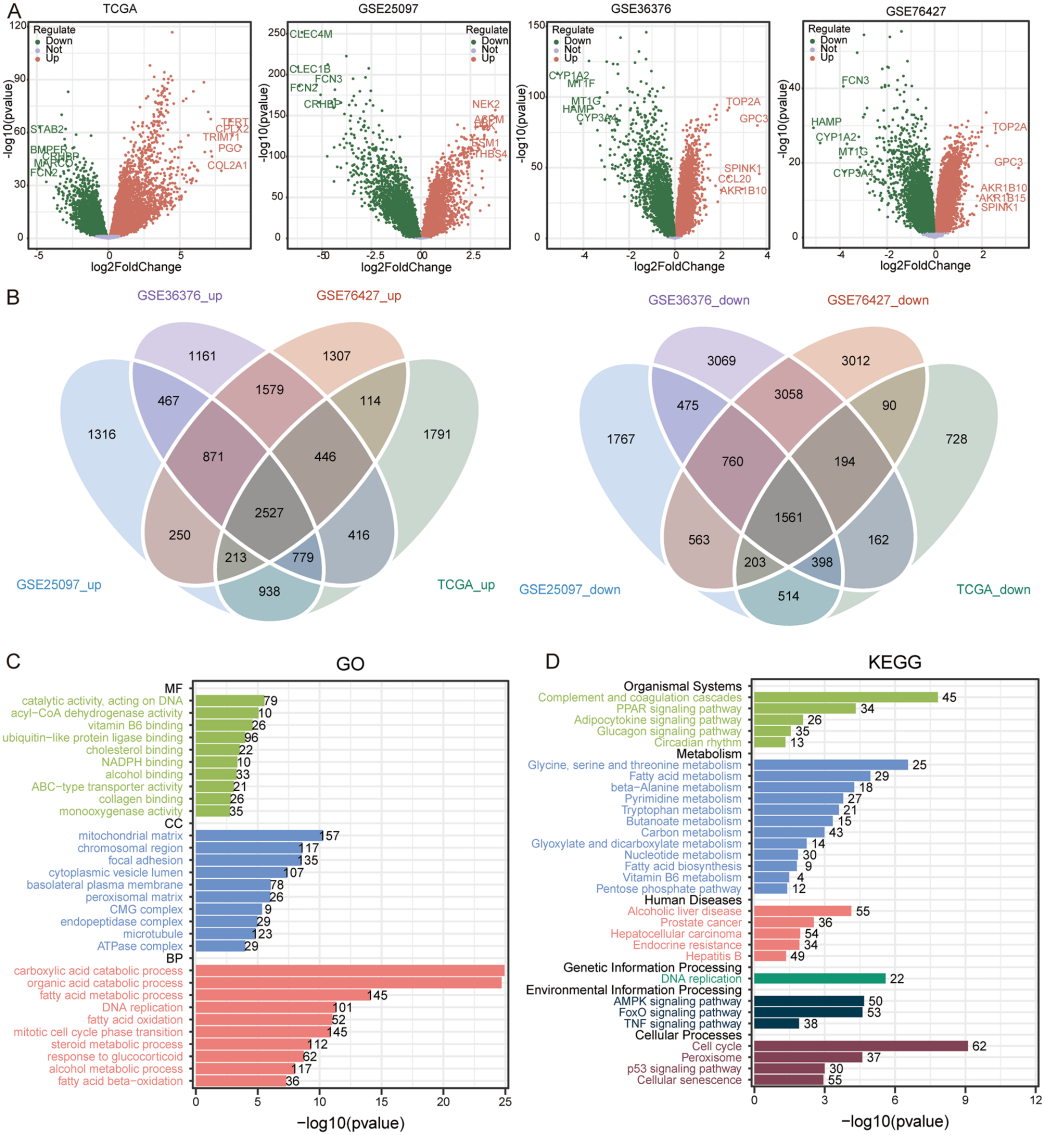
Identification of differentially expressed genes and their participating biological roles. (**A**) The differentially expressed genes in TCGA, GSE25097, GSE36376, and GSE76427 datasets. Red is upregulated expression and green is downregulated expression in HCC. Genes with the largest fold change are labeled. FC, fold change. (**B**) The intersection of upregulated (left) or downregulated (right) expressed DEGs in four datasets. (**C**) GO enrichment results of common DEGs. *MF* molecular functions, *CC* cellular composition, *BP* biological processes. (**D**) KEGG pathways of common DEGs involved in.

After data processing and differential analysis, we obtained 11,399 DEGs in TCGA, 13,839 DEGs in GSE25097, 19,324 DEGs in GSE36376, and 15,656 DEGs in GSE76427 dataset (Fig. 2A). By filtering for DEGs whose direction of differential expression was consistent in all four datasets, we identified 4088 common DEGs, including 2527 upregulated DEGs and 1561 downregulated DEGs (Fig. 2B). The results of GO enrichment (Fig. 2C) showed that the common DEGs mainly enriched in organic acid catabolic process, and carboxylic acid catabolic process of biological processes, fatty acid metabolic process, and focal adhesion of cellular composition, as well as ubiquitin-like protein ligase binding, and catalytic activity, acting on DNA of molecular functions. In addition, we found that complement and coagulation cascades, carbon metabolism, alcoholic liver disease, DNA replication, FoxO signaling pathway, and cell cycle were main enriched KEGG pathways by common DEGs (Fig. 2D).

### Candidate genes screening based on cell death

**Figure 3:**
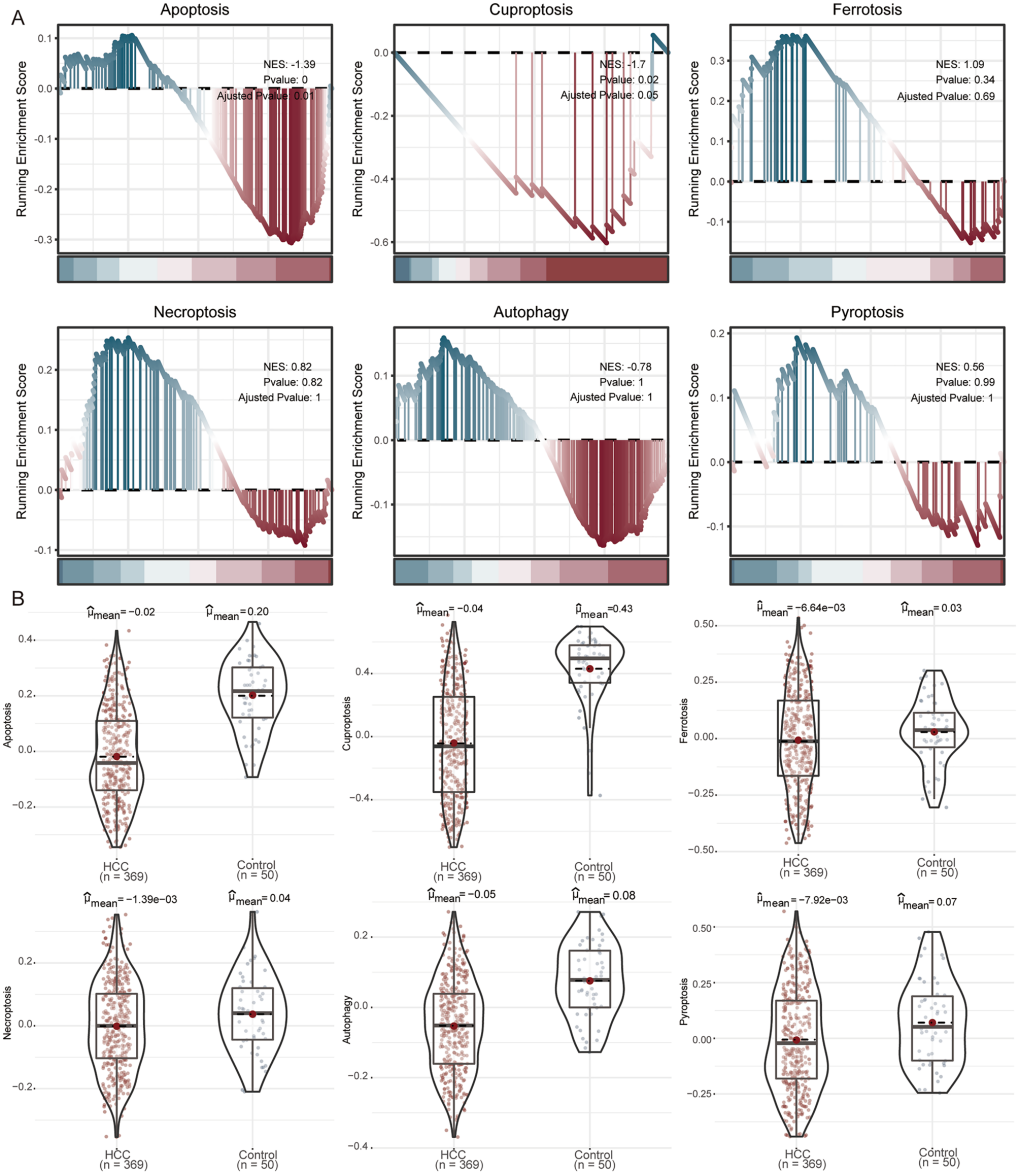
Levels of cell death for HCC in TCGA. (**A**) GSEA of cell death in the HCC. NES, normalized enrichment scores. (**B**) GSVA results of cell death in the HCC. *HCC* hepatocellular carcinoma.

In the TCGA, we used GSEA enrichment to explore the cell death in HCC, including apoptosis, necroptosis, pyroptosis, cuproptosis, ferroptosis, and autophagy. The results showed that apoptosis, and cuproptosis were significantly enriched in controls with higher scores than that in HCC (Fig. 3A). In the results of GSVA, we found that apoptosis was significantly suppressed in HCC (Fig. 3B). Therefore, we chose apoptosis as our focus in subsequent studies.

**Figure 4:**
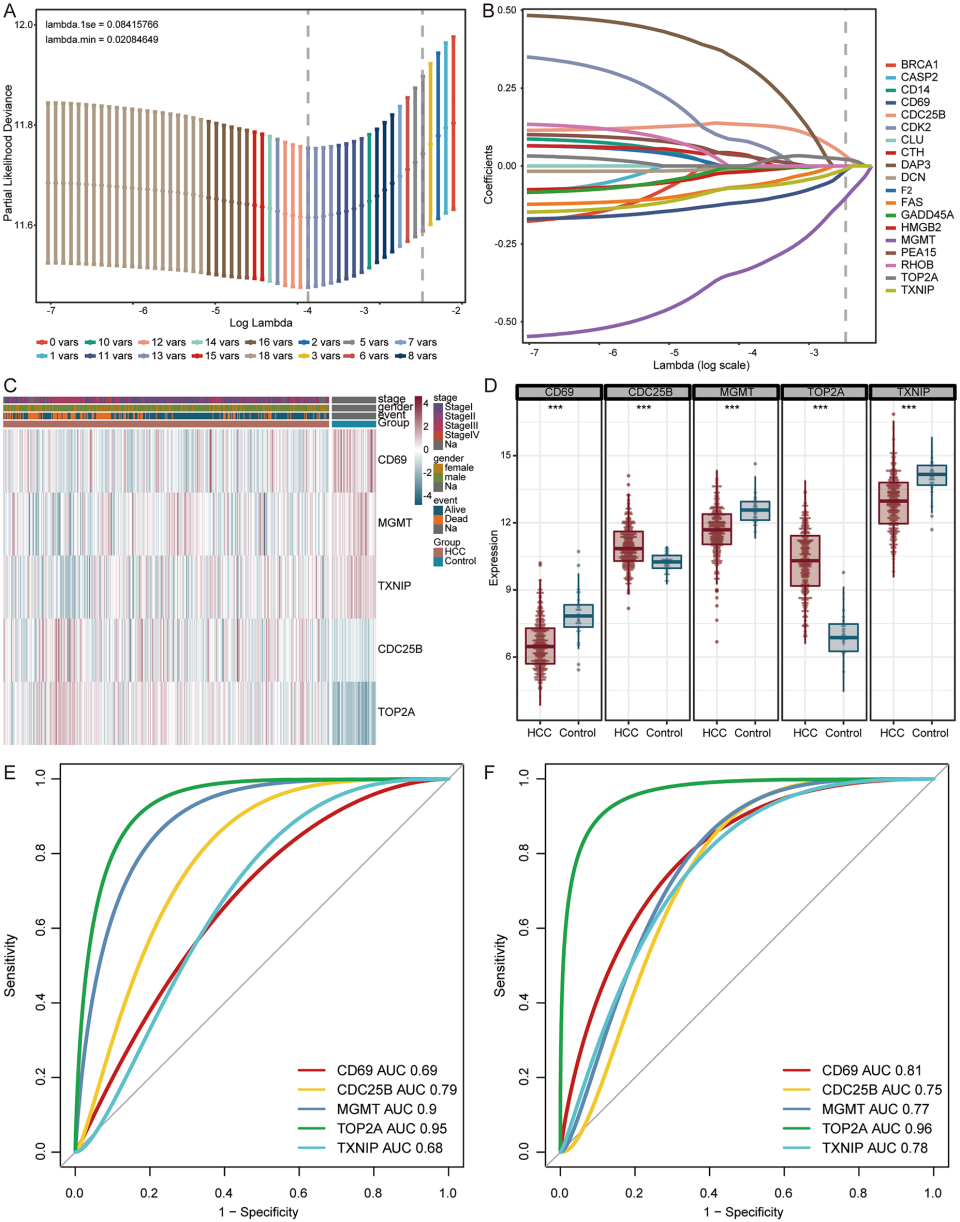
Selection of candidate genes. (**A**) The distribution of LASSO coefficient. (**B**) The optimal parameter selection in the LASSO model. Heatmap (**C**) and box plot (**D**) of expression for candidate genes in TCGA. *HCC* hepatocellular carcinoma. ***P < 0.001. Receiver operating characteristic (ROC) curves of candidate genes in GSE76427 (**E**) and TCGA (**F**).

Furthermore, apoptosis related genes with significant impact on the prognosis of HCC patients were selected for further LASSO regression analysis. Finally, we identified 5 candidate genes with non-zero coefficient (Fig. 4A,B), including CD69, CDC25B, MGMT, TOP2A, and TXNIP. Among them, CD69, MGMT, and TXNIP were downregulated expression in HCC, CDC25B, and TOP2A were upregulated (Fig. 4C,D). ROC curve analyses showed that candidate genes had good sensitive and specific prediction for HCC in GSE76427 (Fig. 4E) and TCGA (Fig. 4F), with all AUC values greater than 0.68.

### Evaluation of candidate genes

**Figure 5:**
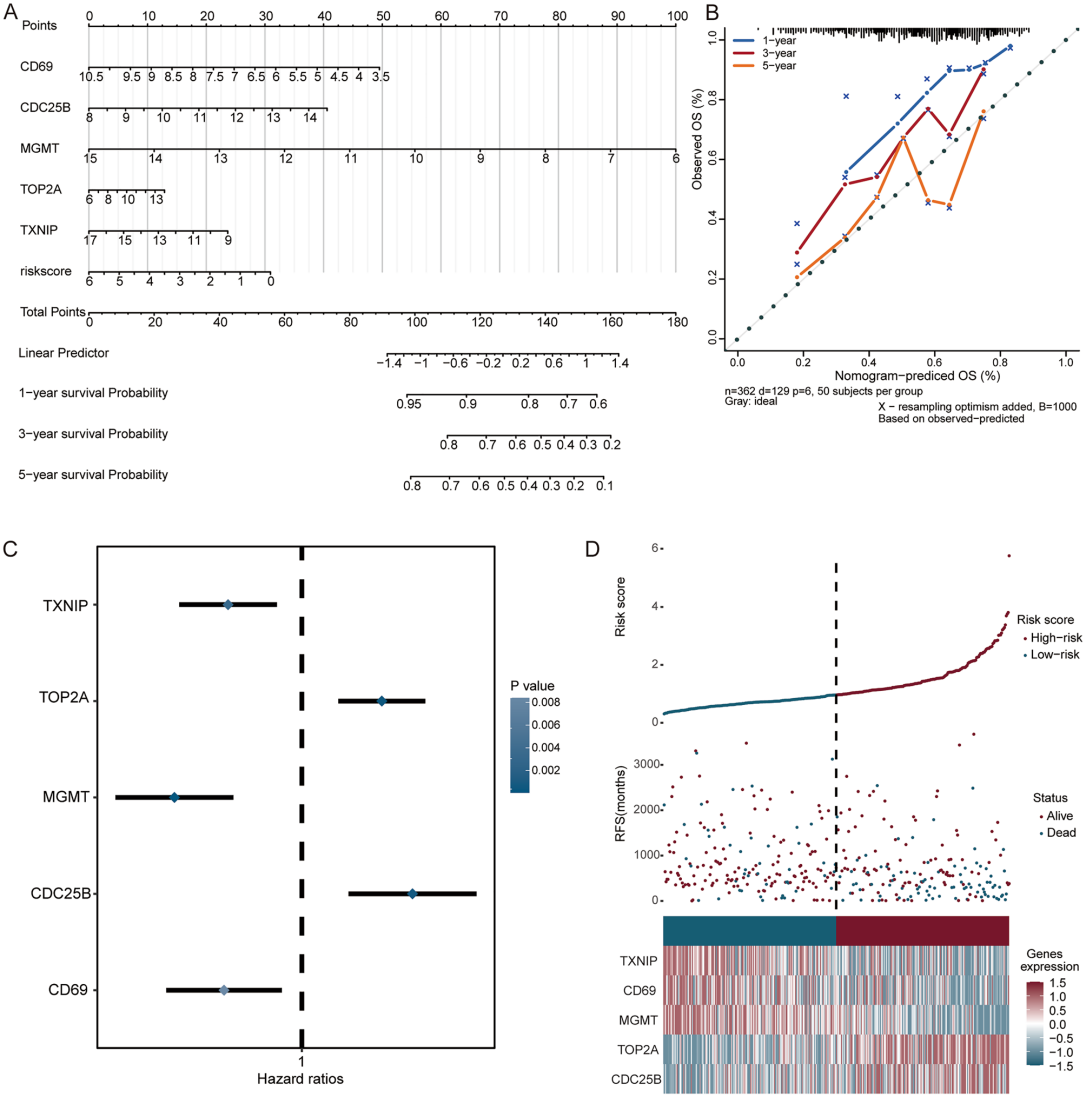
Analysis of candidate genes in TCGA. (**A**) Nomogram of candidate genes to predict the 1, 3 and 5-year survival probability of HCC patients. (**B**) Calibration curves for predicting survival probability of the nomogram. (**C**) Forest plot for the prognostic signature of candidate genes. (**D**) Distribution of risk scores of univariate Cox regression analysis, patient survival durations, and candidate genes expression. *RFS* recurrence-free survival.

We then constructed a prediction model of nomogram for candidate genes to quantify the 1, 3 and 5-year survival probability (Fig. 5A). The calibration curves showed an optimal calibration capability at 1, 3 and 5-year survival probability (Fig. 5B). In the forest plot, we found that CD69, MGMT, and TXNIP were protective factors (hazard ratios < 1), while CDC25B, and TOP2A were risk factors (Fig. 5C).

In addition, according to the risk score in univariate Cox regression analysis, HCC samples were divided into low- and high-risk groups (Fig. 5D). CDC25B, and TOP2A were higher expression, while CD69, MGMT, and TXNIP were lower expression in high-risk group than low-risk group.

### CD69 and Immune infiltration in HCC

**Figure 6:**
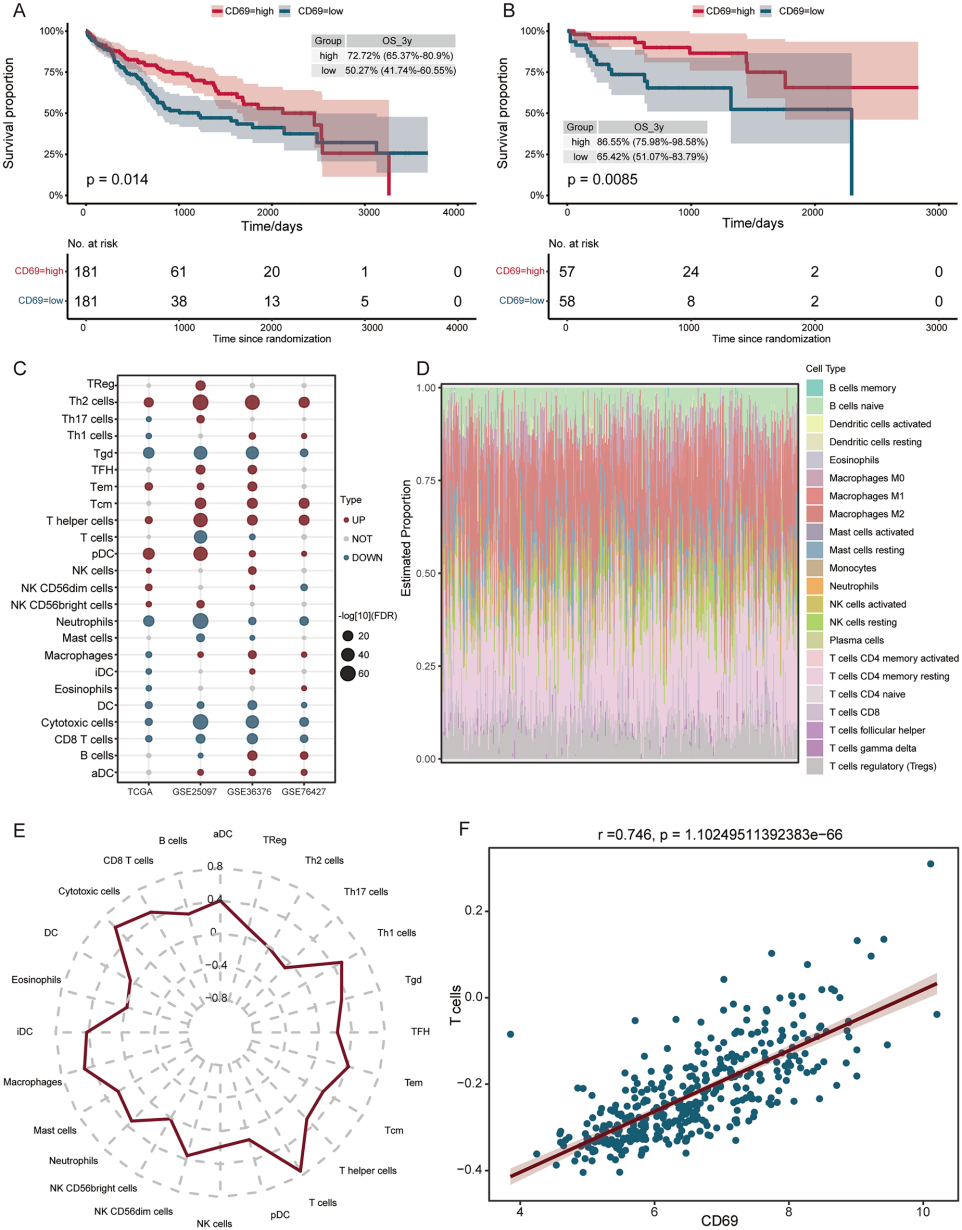
Identification of key gene and immune infiltration in HCC patients. K–M survival curves for CD69 in TCGA (**A**) and GSE76427 database (**B**). (**C**) Significantly high infiltrating (red) or low infiltrating (blue) immune cells in HCC compared to controls in TCGA, GSE25097, GSE36376, and GSE76427 datasets. (**D**) Estimated proportions for immune cells in HCC. (**E**) Correlation between CD69 and immune cells. (**F**) Correlation between CD69 and T cell.

Moreover, we found that CD69 significantly influenced the OS of HCC patients in both TCGA (Fig. 6A) and GSE76427 (Fig. 6B), and then was identified as key gene. Patients with high expression of CD69 with a better outcome. To further analyze the immune infiltration in HCC, we performed ssGSEA and found that T helper 2 (Th2) cells, T helper cells, and plasmacytoid dendritic cells (pDC) were higher infiltration in HCC in all four datasets, gamma delta T cells (Tgd), Neutrophils, DC, cytotoxic cells, and CD8 T cells were lower infiltration in HCC (Fig. 6C). In addition, Macrophages M2, and T cells CD4 memory had more estimated proportion among immune cells (Fig. 6D). Importantly, correlation analysis results showed the highest correlation between CD69 and T cells (Fig. 6E,F).

**Figure 7:**
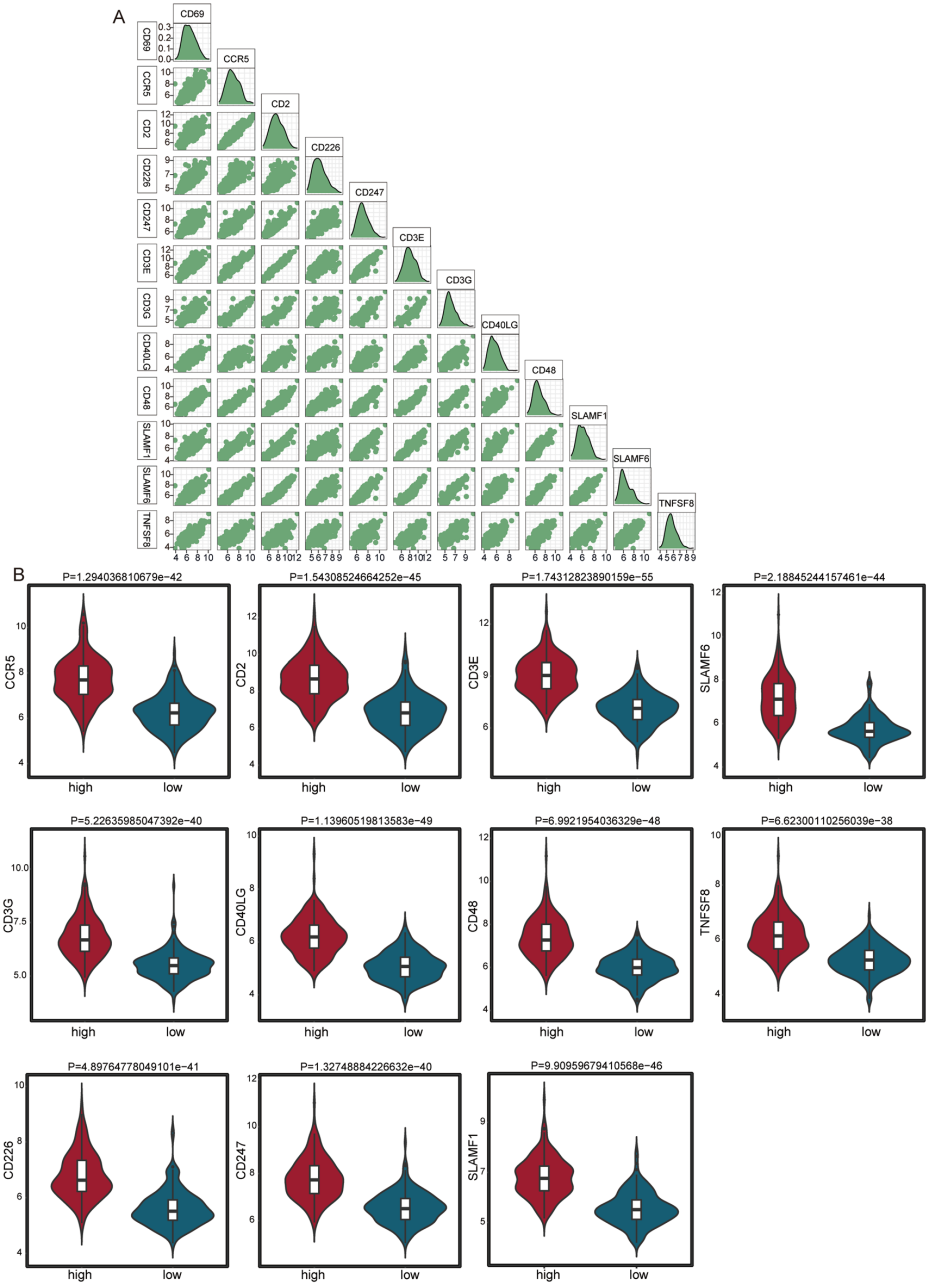
CD69 and immune checkpoints in HCC. (**A**) The correlations between CD69 and immune checkpoints. (**B**) Significantly differentially expressed checkpoints between high and low expression of CD69.

We also calculated the correlation between CD69 and immune checkpoints (Fig. 7A). CD69 and CD3E had the biggest positive correlation. After dividing HCC samples into high and low groups by median expression value of CD69, we found that immune checkpoints were significantly high expressed in high group than low group (Fig. 7B).

## Discussion

Different lethal subroutines during regulated cell death affect cancer progression and response to therapy. In this study, we evaluated aberrant cell death using gene expression profiling data of HCC patients from public databases. The prognostic and diagnostic roles of apoptosis related genes significantly affecting patient survival were identified, and the relationships between key genes and the immune microenvironment were further evaluated. The present study identifies CD69 as a bridge between apoptosis and immunity from the perspective of the mode of cell death in HCC, which participating in pathogenesis and potentially serving as a diagnostic target.

According to the DEGs in HCC among the four datasets, inflammation, metabolism, FoxO signaling pathways, and cell cycle pathways were significantly enriched. Current studies suggest that inflammation can lead to cellular DNA damage, then leading to tumorigenesis^28^. The accumulation of immune cells according to inflammation, resulting in tissue remodeling or impaired function^29^. Immunity and inflammation are the tumor microenvironment, and inflammation will promotes immune responses, leading to immunosuppression^30^. Studies showed that metabolic reprogramming may be a marker for HCC, as liver is an important organ for systemic metabolism^31^. Metabolic disorders such as tyrosine and aspartate have been found in HCC patients and contribute to malignant progression^32,33^. FoxOs are master regulators that determine tumor growth, metastasis, and other characteristics and play important roles in the proliferation, invasion, and metastasis of HCC cells^34,35^. FoxO-Smad complexes can block G1/S transition by regulating the activity of cyclin D and CDK axis^36^. FoxO1 regulated the G1/S checkpoint and apoptosis and functioned as tumor suppressor^37^.

To further explore the cell deaths modalities in HCC, we performed GSEA and GSVA and finally identified that apoptosis was significantly suppressed in HCC. Since cell cycle and cell death exert significant effects on tumor growth, they serve as tumor therapeutic targets^38^. By bioinformatics analysis, we identified CD69, CDC25B, MGMT, TOP2A, and TXNIP as candidate genes. CD69, MGMT, and TXNIP were downregulated expression in HCC and may have protective roles for outcome of HCC patients.

CD69 was identified as a key gene as it significantly impacted patient overall survival in TCGA and GSE76427. Increased the number of CD69+CD8+ T cells to enhance antitumor efficacy in mouse model of HCC^39^. CD69+CD8+T cells were predictor for HCC prognosis, and patients showed significantly longer OS time with expressing higher CD69_ CD8a signaling^40^. CD69+ activated CD8+ T cells are significantly reduced in diethylnitrosamine induced HCC models and participate in tumor evasion of immune responses^41^. MGMT plays an important effector molecule in the DNA damage repair, and its overexpression restores U0126 induced chemosensitivity for temozolomide of HCC cells^42^. MGMT has a poor patient prognosis when it is downexpressed in HCC cells and tumors^43^. The main function of TXNIP is to induce apoptosis under oxidative stress and also to inhibit the proliferation and migration of cancer cells^44^. TXNIP is suppressed in HCC patients, acts as a negative regulator of aerobic glycolysis during HCC progression, and is associated with a worse prognosis^45^.

CDC25B, and TOP2A were upregulated expression in HCC and may have risk roles for HCC. CDC25B is a cell cycle activated phosphatase that positively regulates cyclin dependent kinase activity and is significantly more expressed in HCC than in non-tumor liver^46^. CDC25B has oncogenic properties that enhance tumor growth and survival^47^. The expression level of upregulated CDC25B is negatively correlated with patient survival^48^. TOP2A is upregulated in HCC, which promotes epithelial mesenchymal transition thereby enhancing the metastasis of HCC^49^. Overexpression of TOP2A promoted HCC proliferation and metastasis and was negatively correlated with patient prognosis^50,51^. Moreover, high expression of TOP2A gene can predict poor prognosis after curative resection of HCC^52^. These results suggest that candidate genes may be potential factors for predicting HCC prognosis and potential therapeutic targets.

On the other hand, although immunotherapy was shown to be beneficial for survival in some patients, response rates were generally disappointing and treatment failure may be attributed to the strong immunosuppressive tumor microenvironment in the liver^53^. Hepatic γδ T cells exhibit typical liver tissue tropism by expressing CD69. In the absence of dependence on T cell receptor signaling, γδ T cells can directly exert cytotoxic activity to participate in anticancer immunity^54^. CD69 is significantly and positively correlated with T cells and may suppress immune responses in HCC. These results suggest that elevating CD69 may change immunosuppressed tumors to an immune state with better prognosis, which may facilitate HCC immunotherapy.

Our study also has some limitations. First and importantly, the data analyzed are derived from public databases, and clinical availability of key results needs verification in a large number of clinical samples, especially the lack of information related to hepatitis B virus and hepatitis C virus infection. Second, whether CD69, as an apoptosis related gene, is involved in the pathological mechanism of HCC through its regulation on T-cell apoptosis remains unknown. Although we screened candidate genes through LASSO regression analysis, no further experimental validation was conducted, such as functional experiments or molecular mechanism studies. The methods we usen in this study can reveal potential associations and trends, they cannot directly prove causal relationships. In addition, for the key results obtained in this study, further in-depth studies combining bioinformatics and molecular experiments are required, which will benefit the design and development of safe and effective means for HCC treatment.

## Conclusion

In conclusion, we confirmed that low expression of CD69 contributed to the poor prognosis of HCC patients, which may be associated with apoptosis and immunosuppression. Our results provide new insights into the functions of apoptosis related genes, which may be used as potential references for drug targets and accurate survival prediction in patients with HCC disease.

## Data Availability

All data generated or analyzed during this study are downloaded from TCGA, GSE25097, GSE36376, and GSE76427 datasets.

## Abbreviations

AUC: Area under the receiver operating characteristic curve
DEGs: Differentially expressed genes
Tgd: Gamma delta T cells
GEO: Gene Expression Omnibus
GO: Gene Ontology
GSEA: Gene set enrichment analysis
GSVA: Gene set variation analysis
HCC: Hepatocellular carcinoma
K–M: Kaplan–Meier
KEGG: Kyoto Encyclopedia of Genes and Genomes
LASSO: Least absolute shrinkage and selection operator regression
OS: Overall survival
pDC: Plasmacytoid dendritic cells
ssGSEA: Single‐sample GSEA
Th2: T helper 2
